# Asthma and its relationship with anthropometric markers among adults

**DOI:** 10.1371/journal.pone.0281923

**Published:** 2023-02-17

**Authors:** Khalid S. Alwadeai, Saad A. Alhammad

**Affiliations:** Department of Rehabilitation Science, College of Applied Medical Sciences, King Saud University, Riyadh, Saudi Arabia; SMS Medical College and Hospital, INDIA

## Abstract

**Background:**

Many studies have examined the association between anthropometric indicators and the likelihood of developing asthma. However, no study has yet examined the link between asthma and anthropometric markers of risk. This study addresses this gap in the literature by evaluating the relationship between asthma, smoking, and anthropometric measurements such as body mass index (BMI), waist circumference (WC), hip circumference (HC), and waist-to-hip ratio (WHR) among individuals residing in the United States.

**Methods:**

This cross-sectional study conducted a secondary analysis of the 2011–2014 National Survey of Midlife Development in the United States, using data from 2,257 participants aged 25–74. We classified the participants into four groups based on self-reported smoking and asthma status: nonsmokers with no asthma, asthma alone, smokers only, and smokers with asthma. The outcomes of interest were BMI, WC, HC, and WHR scores in the latter three groups compared to the nonsmokers with no asthma group.

**Results:**

Linear regression analysis showed that those with asthma alone and smokers with asthma were significantly more likely to have a BMI, WC, or HC score of 1 or higher than people without asthma and smokers only.

**Conclusion:**

A higher score on the anthropometric parameters was substantially related to participants who had only asthma and those who had both asthma and smoking.

## Introduction

Obesity and asthma are common illnesses that pose substantial risks to public health [1]. Social and environmental risk factors can contribute to the development of both diseases [2]. Recent studies have identified possible connections between these illnesses, which have seen parallel growth globally [2]. Specifically, obesity has been linked to a higher risk for asthma, and 38% of Americans who have asthma at present also have obesity [3].

Although the causes of obesity in people with asthma are not well known, recent research has identified multiple factors [2]. For example, oral corticosteroids may increase lipid uptake and storage in the peripheral tissue, or they may increase insulin resistance. In addition, the reduction of physical activity in people with asthma due to recurrent dyspnea may contribute to an increased body weight [2, 4].

The unidirectional relationship between obesity and asthma has been extensively investigated. However, recently, a recent paradigm shift has occurred, and asthma is now considered a risk factor for obesity development [2, 4]. One recent study provided insight into this inverse association in adults: in European nations, adults with asthma are more likely to become obese than adults without it [2]. That study employed BMI as the sole epidemiological indicator for obesity. However, this marker alone cannot explain the distribution of body fat [5, 6], as physiological variations may influence the clinical symptoms of obesity in the distribution of adipose tissue [7].

Obese smokers with asthma have lower quality of life and use more medical resources [3, 8, 9] than nonobese smokers with asthma. The literature indicates that body fat and smoking increase the risk for asthma, pulmonary dysfunction [10], and quality of life two- or threefold [11, 12]. In addition, smokers with asthma exhibit poor asthma control and have rapidly deteriorating airways, which leads to lung function reduction [13]. As a result, they become less active, which in turn impacts obesity markers. This behavioral variable, which is shared by people with asthma and those with obesity, must be considered.

A retrospective study indicated that obesity is associated with asthma development in a Latino cohort of girls [14]. Although the combination of obesity and asthma is prevalent in the United States [15–17], and it is influenced by the factors of ethnicity, age, and sex [18], the relationship between asthma, and obesity in this new paradigm has not been explored, a lack that is all the more apparent in adult populations. This study fills this gap in the literature by examining the associations among smoking, asthma, and anthropometric indicators, such as BMI, WC, HC, and WHR, among adults in the United States. We predicted that smoking, asthma, or both would increase the likelihood of having a high BMI, WC, HC, and WHR.

## Materials and methods

In this cross-sectional study, we used data from the publicly available Midlife Development in the United States (MIDUS) refresher datasets to conduct a secondary analysis [19]. The MIDUS refresher, a nationwide probability study, involved 3,577 people between the ages of 25 and 74 in an interdisciplinary project examining psychosocial factors and people’s health by gathering demographic and biomarker data during an initial structured telephone interview and clinic visit, respectively [20]. A weighted response rate for the telephone interview and clinic visit was applied to all respondents across sample types to generate unbiased estimates that incorporated the abovementioned variables in the analyses. The procedures used for recruitment and evaluation in the MIDUS refresher are provided elsewhere [19].

We accessed the data in conformity with the privacy and protection policies of the National Archive of Computerized Data on Aging (NACDA). The institutional review boards (IRBs) of Harvard University, Georgetown University, the University of California in Los Angeles, and the University of Wisconsin approved the MIDUS refresher. All of the participants in the study provided written informed consent. The King Saud University IRB’s ethical committee exempted the current study (E-22-6855).

We analyzed data from 2,257 persons aged between 25 and 74. Smoking status was assessed with the following question: “Do you smoke cigarettes regularly?” Asthma was assessed using the following questions: “In the past twelve months, have you experienced, or been treated for asthma?” The participants were classified as nonsmokers with no asthma if they answered “no” to both questions. Participants who answered the first question “no” and the second question “yes” were classified as having asthma alone, while those who answered the first question “yes” and the second question “no” were classified as smokers alone. Participants who answered “yes” to both questions were defined as smokers with asthma. Self-reported smoking status and asthma have been evaluated in prior studies and have been shown to be valid for adults [21].

The outcomes of interest were scores for the anthropometric markers BMI, WC, HC, and WHR in the four groups of participants. BMI was computed by dividing the participants’ weight in kilograms (kg) by their height in meters (m) squared. The participants’ height (in inches) was multiplied by 0.02 to obtain the height in meters. The participants’ weight (in pounds) was multiplied by 0.45 to acquire the mass in kilograms. We set the height to 84 inches if it exceeded 84 inches in limiting extremes. We assessed WC using the following question: “What is your waist size—that is, how many inches around is your waist?” We instructed the participants to measure their waists at the level of their navel at the largest point.

We assessed the HC using the following question: “What is your hip size—that is, how many inches do your hip measurement at the widest point? Measure at the vastest point between your waist and your thigh.” The participants were instructed to answer these questions by measuring themselves while standing using non-stretchable tape. They were requested to avoid measuring over clothing, including thin clothing and to try to record answers to the nearest quarter (1/4) inch. We calculated the participants’ WHR by dividing the WC (in inches) by the HC (in inches).

The covariates age, sex, race, education, marital status, employment, and alcohol intake were included. We dichotomized all of these collected variables as <65 years (reference) and ≥65 years, male and female (reference), white (reference) and minority (Black, mixed, Asian, and others), school/college and graduates (reference), married (reference) and unmarried/divorced/widowed, employed and unemployed (reference), and alcohol consumption (yes [reference] and no).

We used the Farrington-Manning test to calculate the necessary sample size per group to establish valid results using the level of significance (alpha = .05), power (.8), and proportion between groups (0.32, 0.21) [22]. The necessary minimum sample per group was 150. We utilized a Shapiro-Wilk test to determine data normality [23]. Descriptive statistics were established for the constant and definite variables, including means (standard deviations) and number (percentage). We provided the average distributions of BMI, WC, HC, and WHR for all four groups. We used a chi-square test for categorical variables, and independent Student’s t-tests were used for continuous variables to compare groups.

We used linear regression models to investigate the relationships between asthma, smoking, and anthropometric markers, including BMI, WC, HC, and WHR. Model 1 accounted for all four groups and each anthropometric marker. Model 2 featured adjustments for age, sex, race, education, marital status, employment, and alcohol intake in addition to model 1. We presented each model’s estimates (β) and the corresponding standard errors (SE). The nonsmokers with no asthma were used as a reference. We performed all statistical analyses using Stata 14.1 statistical software (Stata Corp, 2015). Statistical significance was defined as *p* < .05.

## Results

**Fig 1:**
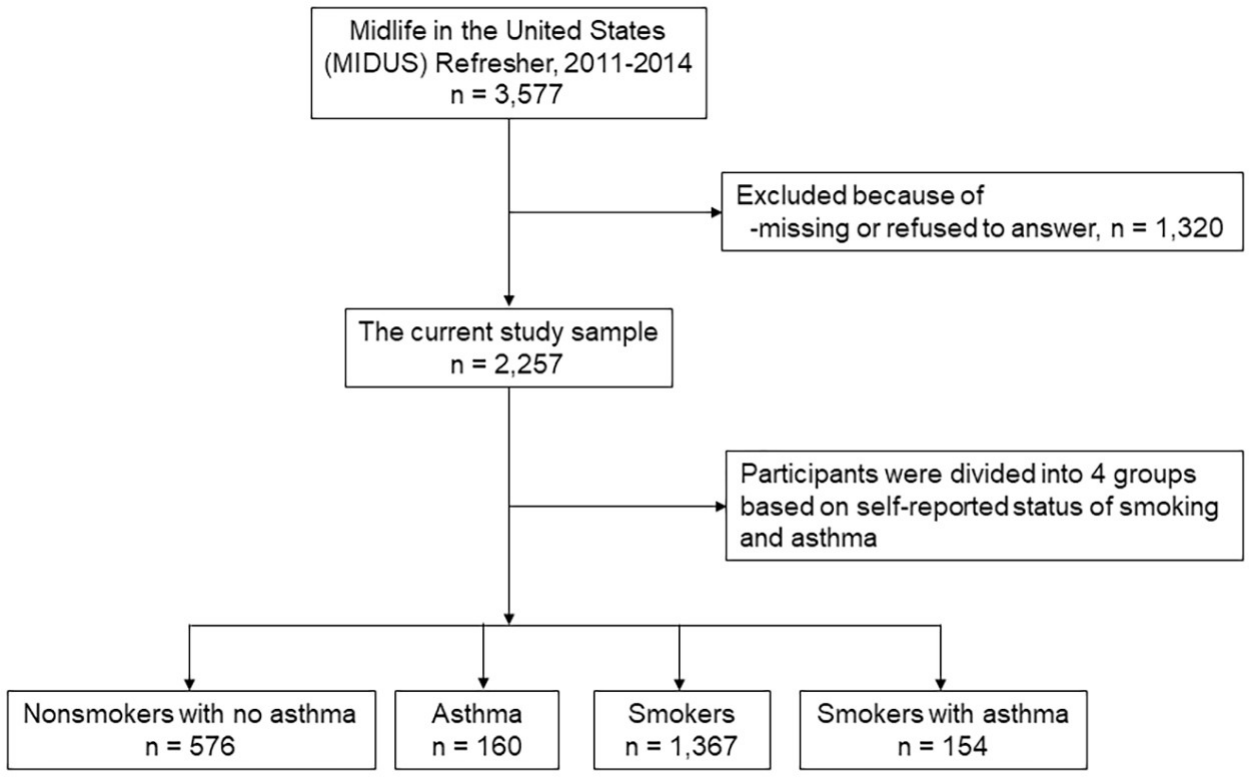
Flowchart of the study participants.

We included data from 2,257 individuals of 3,577 total investigated after excluding 1,320 persons with missing data (Fig 1). Smokers with asthma were 5 years older than participants of the other three groups. Most participants in the groups of smokers and nonsmokers without asthma were under 65 years old. About 63.6% of the male respondents were smokers with asthma. Individuals who smoked and had asthma were more likely to have completed at least some college education (72.7%), and their BMI, WC, HC, and WHR scores were higher (Table 1).

**Table 1:** Characteristics of the study sample.

Characteristics	Nonsmokers with no asthma n = 576 (25.5%)	Asthma n = 160 (7.1%)	Smokers n = 1,367 (60.6%)	Smokers with asthma 154 (6.8%)	*P-value*
Age in years, mean (SD)	50.4 (14.0)	51.6 (14.4)	52.7 (14.5)	57.7 (13.0)	*<* .*0001*
Age group, n (%)					.*003*
< 65 years	456 (79.2)	121 (75.6)	989 (72.3)	98 (63.6)	
≥ 65 years	120 (20.8)	39 (24.4)	378 (27.7)	56 (36.4)	
Sex, n (%)					*<* .*0001*
Male	287 (49.8)	107 (66.9)	628 (45.9)	98 (63.6)	
Female	289 (50.2)	53 (33.1)	739 (54.1)	56 (36.4)	
Height in inches	5.6 (3.4)	5.0 (3.0)	5.3 (3.4)	5.4 (3.1)	.*132*
Weight in pounds	183 (48)	198 (51)	187 (44)	190 (47)	.*002*
Race, n (%)					.*011*
White	496 (86.7)	119 (76.3)	1,161 (85.2)	127 (82.5)	
Minorities*	76 (13.3)	37 (23.7)	202 (14.8)	27 (17.5)	
Educational level, n (%)					*<* .*0001*
School/college	228 (39.6)	77 (48.1)	907 (66.4)	112 (72.7)	
Graduates	348 (60.4)	83 (51.9)	458 (33.6)	42 (27.3)	
Marital status, n (%)					*<* .*0001*
Married	406 (70.7)	91 (56.9)	809 (59.3)	72 (46.7)	
Unmarried/divorced/widow	168 (29.3)	69 (43.1)	555 (40.7)	82 (53.2)	
Employment status, n (%)					*<* .*0001*
Employed	403 (70.1)	104 (65.8)	820 (60.1)	71 (46.1)	
Unemployed	172 (29.9)	54 (34.2)	545 (39.9)	83 (53.9)	
Alcohol intake, n (%)					*<* .*0001*
Yes	113 (19.8)	53 (34)	206 (22.2)	50 (32.7)	
No	459 (80.2)	103 (66)	722 (77.8)	103 (67.3)	
Body mass index in kg/m^2^	28.0 (7.3)	31.9 (8.5)	29.0 (6.3)	30.7 (7.4)	*<* .*0001*
Waist circumference in inches	37.4 (6.8)	39.7 (6.7)	38.9 (6.6)	40.3 (7.4)	*<* .*0001*
Hip circumference inches	40.5 (6.6)	43.2 (6.8)	41.1 (6.4)	42.7 (7.9)	*<* .*0001*
Waist-to-hip ratio	0.93 (0.2)	0.92 (0.1)	0.95 (0.1)	0.96 (0.1)	.*049*

*Black, mixed, Asian, and others.

Compared to nonsmokers with no asthma, individuals with asthma, smokers, and smokers with asthma were significantly associated with increased BMI scores of 31.3, 29.3, and 30.7 kg/m^2^, respectively. After controlling for confounders, all three groups were still significantly associated with increased BMI scores of 31.9 kg/m^2^ and 30.7 kg/m^2^, respectively. The adjusted model increased the explained variance (R^2^ .04 to .09) for those with asthma alone, .007 to .054 for smokers, and .021 to .076 for smokers with asthma than the unadjusted model (Table 2).

**Table 2:** Associations of asthma and smoking with body mass index.

Variable	Model 1			Model 2		
	*β*	SE	*P*	*β*	SE	*P*
**No asthma and nonsmokers**	29.2	0.26	*<* .*0001*	28.8	0.35	*<* .*0001*
Asthma	2.1	0.36	*<* .*0001*	1.4	0.36	.*001*
*R* ^*2*^	.04			.09		
**No asthma and nonsmokers**	28.5	0.20	*<* .*0001*	28.4	0.26	*<* .*0001*
Smokers	0.81	0.23	.*007*	0.45	0.24	.*063*
*R* ^*2*^	0.007			.05		
**No asthma and nonsmokers**	28.9	0.28	*<* .*0001*	28.5	0.39	*<* .*0001*
Asthma and smokers	1.8	0.45	.*001*	1.05	0.46	.*025*
*R* ^*2*^	.02			.08		

***Abbreviations***: *β*, estimate; SE, standard error.

Model 1: unadjusted

Model 2: adjusted for age, sex, race, education, marital status, employment status, and alcohol intake

Individuals with asthma, smokers, and smokers with asthma reported considerably higher WC scores, 42.9, 41.3, and 42.6 inches, respectively, than nonsmokers without asthma. After adjusting for all covariates, the WC scores of individuals with asthma and smokers with asthma were equal to or higher than 42.5 and 42.2 inches, respectively. Relative to the unadjusted model, the explained variance (R^2^) for people with asthma alone (.03–.08), smokers (.003–.05), and smokers with asthma (.02–.06) increased in the adjusted analysis (Table 3).

**Table 3:** Associations of asthma and smoking with waist circumference.

Variable	Model 1			Model 2		
	*β*	SE	*P*	*β*	SE	*P*
**Nonsmokers with no asthma**	41.3	0.24	*<* .*0001*	41.4	0.32	*<* .*0001*
Asthma	1.6	0.33	*<* .*0001*	1.1	0.34	.*014*
*R* ^*2*^	.03			.08		
**Nonsmokers with no asthma**	40.8	0.20	*<* .*0001*	41.2	0.26	*<* .*0001*
Smokers	0.55	0.23	.*020*	0.41	0.24	.*09*
*R* ^*2*^	.03			.05		
**Nonsmokers with no asthma**	41.2	0.27	*<* .*0001*	41.3	0.37	*<* .*0001*
Smokers with asthma	1.4	0.43	.*008*	0.95	0.45	.*033*
*R* ^*2*^	.02			.06		

***Abbreviations***: *β*, estimate; SE, standard error.

Model 1: unadjusted

Model 2: adjusted for age, sex, race, education, marital status, employment status, and alcohol intake

Individuals with asthma, smokers, and smokers with asthma had substantially higher HC scores of 40, 39, and 40.2 inches, respectively. After controlling for confounders, individuals in these groups still had significantly higher HC scores, 39.1, 38.2, and 39.2 inches, respectively. The explained variance (R^2^) increased in the adjusted variance from .03 to .10 for those with asthma alone, from .01 to .08 for smokers, and from .03 to .09 for smokers with asthma (Table 4). Smokers alone and smokers with asthma were significantly associated with higher WHR scores of .01, respectively (Table 5).

**Table 4:** Associations of asthma and smoking with hip circumference.

Variable	Model 1			Model 2		
	*β*	SE	*P*	*β*	SE	*P*
**Nonsmokers with no asthma**	38.3	0.24	*<* .*0001*	37.6	0.32	*<* .*0001*
Asthma	1.7	0.33	*<* .*0001*	1.5	0.34	*<* .*0001*
*R* ^*2*^	.03			*<* .*001*		
**Nonsmokers with no asthma**	37.9	0.20	*<* .*0001*	37.5	0.26	*<* .*0001*
Smokers	1.1	0.23	*<* .*0001*	0.73	0.24	.*024*
*R* ^*2*^	.01			.08		
**Nonsmokers with no asthma**	38.3	0.27	*<* .*0001*	37.6	0.36	*<* .*0001*
Smokers with asthma	1.9	0.42	*<* .*0001*	1.5	0.43	.*006*
*R* ^*2*^	.03			.09		

***Abbreviations***: *β*, estimate; SE, standard error.

Model 1: unadjusted

Model 2: adjusted for age, sex, race, education, marital status, employment status, and alcohol intake

**Table 5:** Associations of asthma and smoking with a waist-to-hip ratio.

Variable	Model 1			Model 2		
	*β*	SE	*P*	*β*	SE	*P*
**Nonsmokers with no asthma**	0.93	0.05	*<* .*0001*	0.92	0.07	*<* .*0001*
Asthma	0.06	0.08	.*411*	0.01	0.08	.*202*
*R* ^*2*^	.08			.15		
**Nonsmokers with no asthma**	0.93	0.04	*<* .*0001*	0.91	0.06	*<* .*0001*
Smokers	0.01	0.06	.*014*	0.05	0.05	.*322*
*R* ^*2*^	.01			.13		
**Nonsmokers with no asthma**	0.94	0.06	*<* .*0001*	0.92	0.08	*<* .*0001*
Smokers with asthma	0.01	0.01	.*121*	0.01	0.01	.*229*
*R* ^*2*^	.03			.15		

***Abbreviations***: *β*, estimate; SE, standard error.

Model 1: unadjusted

Model 2: adjusted for age, sex, race, education, marital status, employment status, and alcohol intake

## Discussion

This study investigated the relationships between anthropometric markers in adults, as evidenced by obesity indicators and asthma, smoking, or both. Even after controlling for confounders, smokers with asthma were significantly more likely to have higher BMI, WC, and HC scores than nonsmokers without asthma. A BMI of 29.8 kg/m^2^, a WC of 42.5 inches, and an HC of 39.2 inches were all significantly associated with having asthma alone. The smoker group was substantially related to an HC of 38.2 inches. Additionally, smokers with asthma were on average 5 years older than the other three groups.

Our findings are aligned with those of previous longitudinal epidemiological research conducted in various European nations [2]. The previous study found that those with asthma were more likely to be obese in their old age, irrespective of asthma medications. Our results indicate that asthma may be a risk factor for obesity. The previous study has relied upon BMI solely as an obesity marker. However, while this measurement estimates overall adiposity, it cannot distinguish between variations in body fat distribution. To overcome this limitation, we used other anthropometric markers and accounted for smoking as a modifiable lifestyle factor.

Importantly, our study found no significant correlation between smoking and BMI after controlling for all confounders. This may have been because smoking has a negligible effect on weight development (averaging only 0.7 kg/m^2^) [24]. Additionally, earlier work by Clair and colleagues has revealed that smokers have lower mean BMI scores than nonsmokers [25].

The preliminary findings in our analysis suggest that individuals who have asthma alone and smokers who have asthma are associated with high anthropometric scores, including BMI, WC, HC, and WHR. Our findings, therefore, have significant implications for developing lifestyle strategies that address the obesity indicators in the treatment of asthma. Because BMI changes may impact lung function, therapeutic techniques targeting healthy diet and physical activity could be implemented to treat the progression of obesity and improve lung function. Further, earlier research has demonstrated the value of pulmonary rehabilitation programs (PRPs), which focus on weight loss and tobacco cessation, in treating asthma [26, 27]. The advantages and efficacy of PRPs have been demonstrated in numerous randomized controlled trials [28, 29].

The main strength of this study is that it provided objective measures of anthropometric markers (namely, BMI, WC, HC, and WHR) in place of self-reported data. People frequently misrepresent their height and weight, resulting in a devalued BMI [30, 31]. Additionally, we used a sufficiently large sample size [32]. Nevertheless, this study had some limitations. First, the cross-sectional design made it impossible to establish a causal link between asthma, smoking, and anthropometric indicators. Second, due to the lack of information or a large amount of missing data, we were unable to assess potential confounding factors in relation to the onset of obesity, such as physical activity, dietary, and environmental factors, use of steroids, history of infections, sleep deprivation, history of breastfeeding in infancy, and patient medication history. Third, the potential bias of self-reporting asthma may have led to misclassification of asthma that should be another obstructive respiratory disease, such as COPD [33]. Numerous studies have indicated that asthma–COPD phenotype overlap syndrome frequently leads to undiagnosed asthma [34, 35].

## Conclusions

Individuals with asthma alone and smokers with asthma were significantly associated with higher BMI, WC, and HC scores than nonsmokers with no asthma, even after controlling for confounders. The results of this study apply to adults in the United States, even though the study relied on participants’ self-reported asthma and smoking habits. Additional analyses are advised to assess other confounding variables, such as medical history, physical activity, diet, dietary, and environmental factors, use of steroids, history of infections, sleep deprivation, history of breastfeeding in infancy, and influence of dietary, or environmental factors in the development of obesity.
